# Multi-View Design Patterns and Responsive Visualization for Genomics Data

**DOI:** 10.1109/TVCG.2022.3209398

**Published:** 2022-12-21

**Authors:** Sehi L’Yi, Nils Gehlenborg

**Affiliations:** Harvard Medical School, Boston, MA, USA.

**Keywords:** Responsive visualization, multi-view visualization, genomics, visualization grammar

## Abstract

A series of recent studies has focused on designing cross-resolution and cross-device visualizations, i.e., responsive visualization, a concept adopted from responsive web design. However, these studies mainly focused on visualizations with a single view to a small number of views, and there are still unresolved questions about how to design responsive multi-view visualizations. In this paper, we present a reusable and generalizable framework for designing responsive multi-view visualizations focused on genomics data. To gain a better understanding of existing design challenges, we review web-based genomics visualization tools in the wild. By characterizing tools based on a taxonomy of responsive designs, we find that responsiveness is rarely supported in existing tools. To distill insights from the survey results in a systematic way, we classify typical view composition patterns, such as “vertically long,” “horizontally wide,” “circular,” and “cross-shaped” compositions. We then identify their usability issues in different resolutions that stem from the composition patterns, as well as discussing approaches to address the issues and to make genomics visualizations responsive. By extending the Gosling visualization grammar to support responsive constructs, we show how these approaches can be supported. A valuable follow-up study would be taking different input modalities into account, such as mouse and touch interactions, which was not considered in our study.

## 1 Introduction

The proliferation of digital devices (e.g., smartphones and tablets) led to an increased diversity of input and output modalities, such as displays with different sizes and resolutions and support for new user interactions. This makes the design of visualizations that can be useful across a variety of contexts more challenging. A series of recent studies [1, 2, 21, 30–32, 67] has focused on designing cross-resolution and cross-device visualizations, i.e., responsive visualizations, a concept adopted from responsive web design [16]. Its design space, as well as actual needs of responsive visualization designers, has been explored through surveys and interviews [21, 30]. Another set of studies explored ways to design responsive visualizations based on manual and automatic approaches [21, 31, 32]. While these studies comprehensively explored the area of responsive designs, they mainly focused on visualizations with a single view or a small number of views. Combined with the complexity and size of multi-view design space [10, 17, 18, 41], there are still unresolved questions about how to design responsive multi-view visualizations.

In this paper, we focus on the design of responsive multi-view visualizations for genomics visualization, which is an important but challenging problem. Due to the complexity of genomics data (e.g., multi-modal and multi-focus aspects [42]), visualization plays a key role in the genomics field. Many data portals for large audiences [54, 70] provide built-in interactive visualizations for exploring data, reflecting the importance of visual analytics and visual communication. For example, a sizable number of visitors of the NIH Human BioMolecular Atlas Program (~25% in a typical month) and NIH 4D Nucleome Consortium (~10%) data portals, are using mobile devices^1^. Moreover, while high resolution displays are popular in these days, many hospitals still use old devices [60], making it important to support smaller resolutions.

However, most genomics visualizations do not scale well to smaller resolutions, limiting the accessibility of genomics data for a large audience. For example, our survey results in this paper show that responsiveness is rarely supported in real-world genomics visualization tools, leading to a wide range of usability issues. In addition, according to our survey results, genomics tools commonly combine many types of visualizations, even more than in the general multi-view visualizations [10]. This makes it even more challenging to design responsive genomics visualization given the limited scalability of smaller screens.

We present a reusable and generalizable framework for designing responsive multi-view visualizations focused on genomics data. To gain a better understanding of existing design challenges, we review web-based genomics visualization tools in the wild. By characterizing tools based on a taxonomy of responsive design, we find that responsiveness is rarely supported in existing tools. To distill insights from the survey results in a systematic way, we classify typical view composition patterns, such as “vertically long,” “horizontally wide,” “circular,” and “cross-shaped” compositions. We then identify their usability issues at different resolutions that stem from the composition patterns and discuss approaches to address the issues that need to be resolved to design responsive genomics visualizations. By extending the Gosling visualization grammar for genomics data [42], we show how these approaches can be seamlessly supported in visualization grammars. Since we did not consider different input modalities, such as mouse and touch interactions, follow-up studies will be needed to better support interactive aspects across different devices.

Key contributions of this paper are three-fold:

- The identification of multi-view design patterns and usability issues of genomics visualization tools in varying screen resolutions through a systematic survey (*N*=40);
- The identification of responsive designs for addressing common usability issues; and
- The extension of Gosling for responsive multi-view designs demonstrated with real world examples.

## 2 Related Work

### 2.1 Responsive Visualization

The term “responsive visualization” was introduced in the more recent literature, but similar ideas have been explored in the human–computer interaction and visualization domains even longer. For example, optimizing the layout of graphical user interfaces depending on window sizes has been a popular research topic [45, 46]. In the visualization domain, display scalability [12], one of key design challenges in visualization, is related to responsive designs. Semantic zooming [51]—changing the visual representation of elements depending on the context of visualization—can be considered a form of responsive design. While the change of visual representation with semantic zooming is most commonly triggered by change in scale (i.e., showing detailed glyphs when the visualization is zoomed in very far [52]), some researchers adopted this idea considering the space availability on the screen as a main triggering factor. For example, GazeDx [61] switches between three different visualizations of the same data (i.e., line charts, histograms, and bar charts) depending on the size of its container panel, controlling the level of information granularity conveyed in the visualizations. Similarly, ThermalPlot [62] dynamically shows additional details in their glyph representations when the given region of the visualization has sufficient space. Responsive matrix cells [23] in another example based on a focus+context technique that controls the level of details based on the available screen space. In the area of mobile visualizations, Wu et al. [67] suggested an automatic approach for updating visualizations to a mobile-friendly design by fixing common usability issues found on smaller screens.

More recently, development of responsive visualization approaches is being discussed more frequently in the literature, indicating a greater need for solutions [1, 2, 4, 21, 22, 30, 32]. Andrews and Smrdel [2], for example, explored the potential use cases of responsive designs with bar charts, line charts, scatterplots, and parallel coordinates. Several studies later expanded knowledge on responsive visualizations in terms of the design space and user needs through surveys on web journal responsive visualizations [21, 30] and user interviews [21, 30]. Horak et al. [22] also reviewed design strategies for responsive visualization, such as layouts and encodings. Researchers also explored the interface aspects of designing responsive visualizations [21, 31, 32]. Hoffswell et al. [21] presented a shelf construction-based graphical user interface for authoring responsive visualizations. Kim et al. [32] built an automated method to design responsive visualizations based on constraint programming. Most recently, Kim et al. [31] proposed a declarative visualization grammar for responsive design, as well as a user interface and a recommendation model built on top of the grammar.

While these studies comprehensively explored the area of responsive design, they did not specifically focus on multi-view visualizations. Considering the complexity and large size of the design space for multi-view visualizations [41], there are still many unclear aspects on how to design useful responsive multi-view visualizations. Focused on genomics data, we extract common usability and scalability issues of visualization tools in varying screen resolutions and present approaches to overcome the issues to make genomics visualization responsive.

### 2.2 Multi-View Visualization

Many visualization studies contribute to our current knowledge about the design space for multi-view visualization as well as understanding their usefulness for different visualization types, tasks, and datasets. There are many survey papers that explore the various design options for constructing multi-view visualizations [10, 13, 18, 26, 41]. Javed at al. [26] proposed four operations to compose multiple views: juxtaposition, superimposition, overloading, and nesting. Focused on comparison tasks, Gleicher et al. [18] identified three primitive building blocks to arrange multiple views, i.e., juxtaposition, superposition, and explicit-encoding. These arrangement techniques were revisited later in a meta-review study [41] which further expanded the design space. Another set of studies focused on providing design guidelines based on the results of empirical studies [25, 40, 41, 49, 53]. For example, many controlled user studies are conducted by researchers to evaluate the usefulness of arrangement types for selected tasks [25, 40, 49]. Qu and Hullman [53] conducted a Wizard-of-Oz study to understand how to consistently use visual channels across multiple views. Based on the review of research papers, including controlled users studies, L’Yi et al. [41] discussed trade-offs of using different view-composition techniques and provided practical design guidelines. While the area of multi-view visualization has been explored in-depth, we still have little knowledge on how to change multi-view designs depending on screen resolutions to enable responsive multi-view visualization. In this paper, we adopt design guidelines suggested in the area of multi-view visualization to identify approaches that address common multi-view usability issues that we characterize.

## 3 Nomenclature: View, Track, and Track Group

**Fig. 1.**
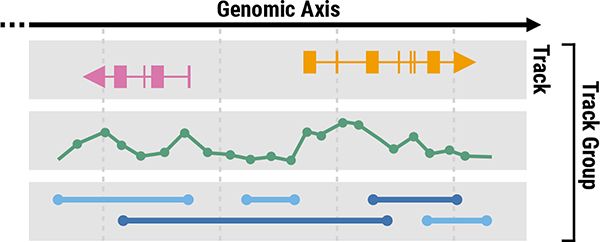
The structure of genomics visualizations with tracks and track groups. A **track** refers to a visualization that can be classified as one of visualization types. A **track group** represents a set of tracks that are aligned to the same genomic axis and coordinated for synchronized navigation with zooming and panning.

Consistent with a survey study on multi-view visualizations [10], we define a **view** as a visualization that can be classified as one of several common visualization types [5], such as bar charts, line charts, and scatterplots. Since our study focuses on genomics data, we additionally consider conventional genomics visualizations as common visualization types [47], such as gene annotations, ideograms, sequence logos [58]. Genomics data visualizations commonly have a nested structure of views, consisting of tracks and track groups. For the purpose of this paper, we use a common genomics term “**track**” [47] to refer to a view. Therefore, a track is the same as a view, and “multi-view” in the paper title is the same as “multi-track.” A track visualizes a single dataset, and up to two of the *x*- and *y*-axes can represent genomic locations, i.e., **genomic axes**. A **track group** represents a set of tracks that are aligned on the same genomic axis for the concurrent analysis of multiple datasets. Genomics visualization tools commonly support synchronous navigation with zooming and panning for multiple tracks that belong to the same group. Fig. 1 illustrates three tracks that use the *x*-axis to represent genomic positions and are aligned and grouped to a single track group. Actual visualization examples can be found in Fig. 5. For example, Fig. 5A shows three track groups each of which contains one or multiple tracks. In the remainder of this paper, we use the terms “tracks” and “track groups” consistently throughout the paper to refer to the structure of genomics visualizations. More details on the background of genomics visualizations can be found in our paper describing the *Gosling* genomics visualization grammar [42].

## 4 Survey of Genomics Visualization Tools

To better understand current challenges of designing responsive multi-track visualizations for genomics data, we reviewed a total of 40 genomics visualization tools in the wild. Through this survey, we identify typical multi-track composition patterns and their usability issues at different screen resolutions.

### 4.1 Method

We first looked into total 188 web-based genomics visualization tools^2^ from two websites that provide large tool collections: GenoCAT [59], an extension of a survey on genomics visualization tools [47], and awesome-genome-visualization [15] which is created and actively maintained by a member of the JBrowse team [8]. For an efficient and focused review process, we excluded the following tools from our survey, reflecting the scope of our research. First, we excluded tools that do not visualize genome-mapped data [42], i.e., tools that do not include visualizations that use a genomic coordinate system (e.g., Metaviz [63]). Second, we excluded tools that do not have working online demos. If a tool provided multiple online demos, we used the first demo from the list in our survey. Third, we excluded visualization libraries (e.g., Circos [36]) unless they provided online demos. We did not consider libraries themselves in our survey because we are mainly interested in reviewing tools for end users, i.e., interfaces with visualizations already composed. However, if a visualization library or framework provided a demonstration-purpose analytics interface (e.g., HiGlass App [28]), we included the interface to our survey. Fourth, since we are mainly interested in multi-track visualizations, we excluded a few tools with only a single track. Lastly, we excluded any duplicated tools (i.e., many tools were found in both collections). After the filtering process, we obtained a list of 40 unique tools. The list of all tools is available in the Supplementary Material.

With the selected 40 tools, we collected the following data:

- Support of responsive designs [30]
- Multi-track design patterns [10]
- Usability issues in varying screen resolutions [67]

First, we wanted to understand to what extent existing genomics visualization tools support responsive designs. To identify responsive designs, we used a taxonomy of responsive visualization [30], while trying to find any other unique designs for genomics visualizations. Second, we collected multi-track design patterns in existing tools, such as the number of tracks and track groups shown in the tools, as well as their composition patterns. We also recorded the dynamic nature of composition patterns, i.e., how tracks and track groups can be added by users on top of previous composition statuses, if tools allowed adding additional tracks. To concisely express and record multi-track composition patterns in our survey, we adopted a tiling algebra [68], which is a set of constraints to express the topology of panels in user interfaces. For example, equations of *A*|*B* and *A*/*B* express that the track *A* and *B* are arranged horizontally and vertically, respectively. To reflect the context of genomics visualizations, we slightly modified the algebra, e.g. to distinguish tracks and track groups and to express repetitions. For example, [*A*]/[*B*/*C*] expresses that three tracks are arranged vertically while the last two tracks are grouped to a single track group. Third, we identified usability issues at different screen resolutions. We adopted the five categories of usability issues that were found in a survey on mobile visualizations [67] and extended them to seven categories to reflect the characteristics of genomics visualizations. These usability categories are illustrated in detail with visual examples in the following sections.

When reviewing each tool, we used five different resolutions that were chosen from previous studies [4, 21, 67]. We used three devices, i.e., Desktop (1920×1080), Google Nexus 9 Tablet (1024×768) [4], and iPhone X (375×812) [21, 67], and two orientations [21] for the two mobile devices, i.e., portrait and landscape. The resolution of each device represents standard *logical resolutions* that are effective in CSS. Following previous studies [21, 67], we used Chrome DevTools^3^ to simulate different devices and orientations when reviewing individual tools. All survey results illustrated in this section are available in the spreadsheet in the Supplementary Material.

### 4.2 Descriptive Results

**Fig. 2.**
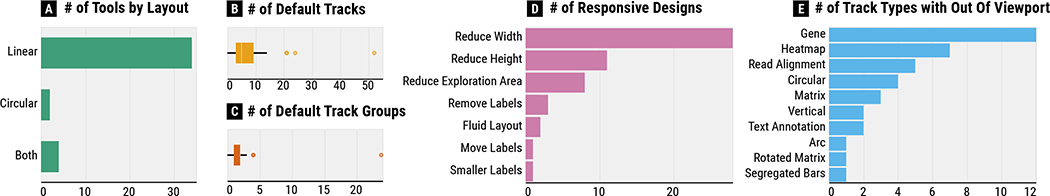
The descriptive survey results: (A) The number of supported layouts, (B) the number of tracks shown by default, (C) the number of track groups shown by default, (D) the number of observed responsive designs, and (E) the number of track types that were unable to see within a screen.

**Fig. 3.**
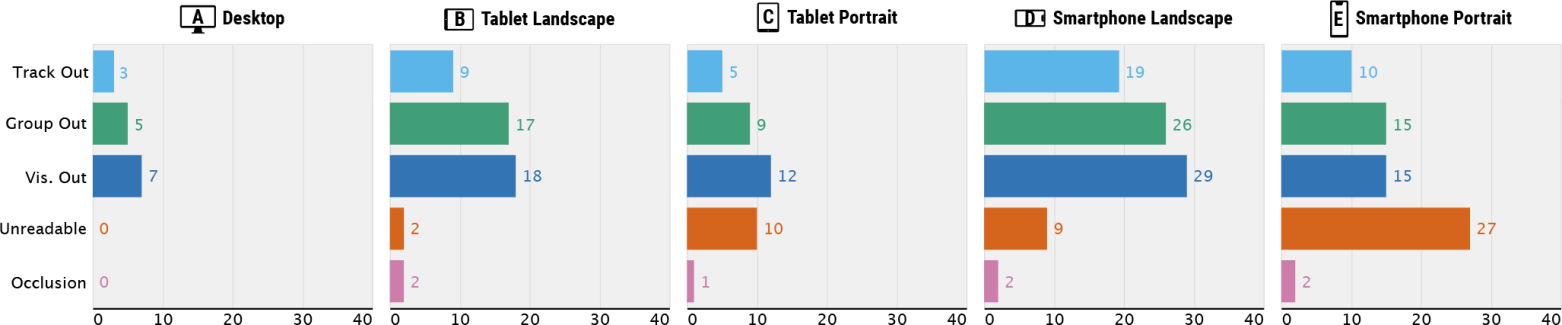
The observed usability issues of genomics visualizations by five different resolutions. Track Out, Group Out, and Vis. Out refer to issues where individual tracks, track groups, and entire visualizations, respectively, become larger than the viewport and cannot be displayed within a screen. Unreadable refers to an issue that marks become too small to interpret. Occlusion refers to an issue where multiple marks overlap.

The summary of descriptive survey results are shown in Fig. 2 and Fig. 3. A majority of tools (85%) displayed visualizations in linear layouts only (i.e., using Cartesian coordinates) while several tools (15%) supported circular layouts as well (i.e., using polar coordinates) (Fig. 2A). The tools showed on average of 8.1 tracks (*SD*=9.4) and 2.4 track groups (*SD*=3.8) by default when a user opens the tools for the first time (Fig. 2B–C). However, more than half of all tools (52.5%) allowed users to add as many tracks as they want on top of the default tracks and track groups. More than half of the tools (55%) provided multiple levels of scale, i.e., showing overviews and detail views.

### 4.3 Lack of Responsive Designs in Genomics Tools

We found very limited support of responsive designs in genomics visualizations tools. When we characterize tools based on a taxonomy of responsive designs [30] (Fig. 2D), we find that seven tools (out of 40) did not support any responsive designs at all. Among the tools with responsive designs, the majority (29 out of 33) supported simple layout changes only, e.g., resizing the width and height of tracks and track groups. Controlling the level of information granularity is considered to be an important factor in responsive designs [30], but this was rarely observed (3 tools that removed labels). The non-layout related responsive designs (4 tools) are all related to text labels only in individual tracks, such as removing, relocating, or resizing them. Notably, none of the genomics tools changed arrangement of views across different screen resolutions (e.g., “serialize layout” in the taxonomy [30]). This indicates that although genomics visualization commonly contains multiple tracks, responsiveness in terms of multi-track design patterns is neglected. In addition, we barely found responsive designs that update data (e.g., filter certain categories) or encoding (e.g., alter to more compact visual representations), which are other common responsive designs according to surveys [21, 30]. Overall, our survey results show that there is substantial need to improve the support of responsive design for genomics visualizations.

### 4.4 Typical Track Composition Patterns

**Fig. 4.**
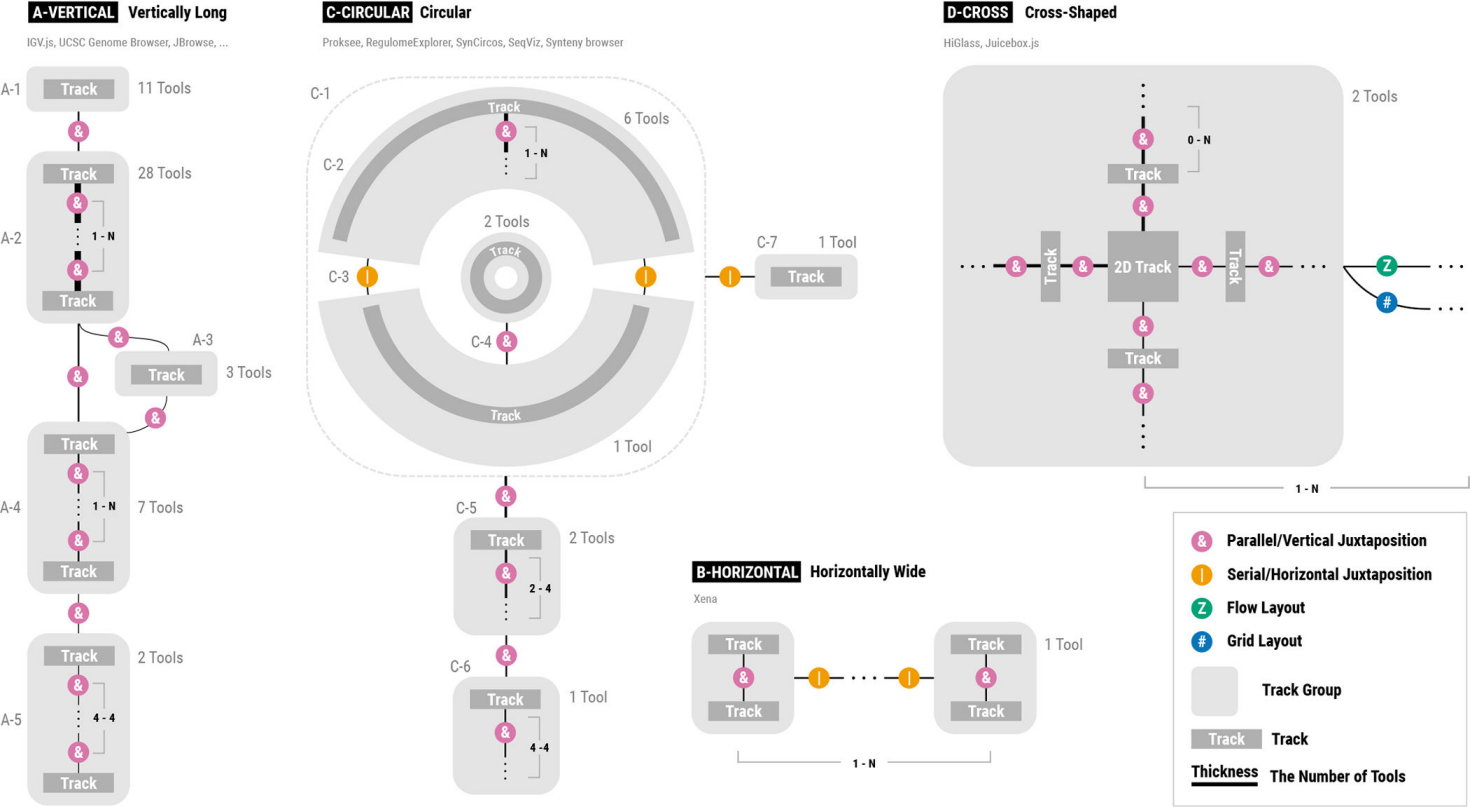
A visual summary of four typical track composition patterns. The light and dark grey rectangles (or arcs) represent tracks and track groups, respectively. Circles with four different colors represent different arrangement types. Three black dots between circles represent repetitions while a pair of numbers in a nearby grey ruler represents minimum and maximum numbers of tracks and track groups that can be composed together in the given tools. These repetitions visually illustrate the dynamic nature of genomics tools, i.e., how additional tracks are composed with the previous composition status. Thicker edges represent a higher number of supporting tools. The grey number next to each of track groups represents the number of supporting tools. The examples of actual visualizations that correspond to individual composition patters are shown in Figure 5.

**Fig. 5.**
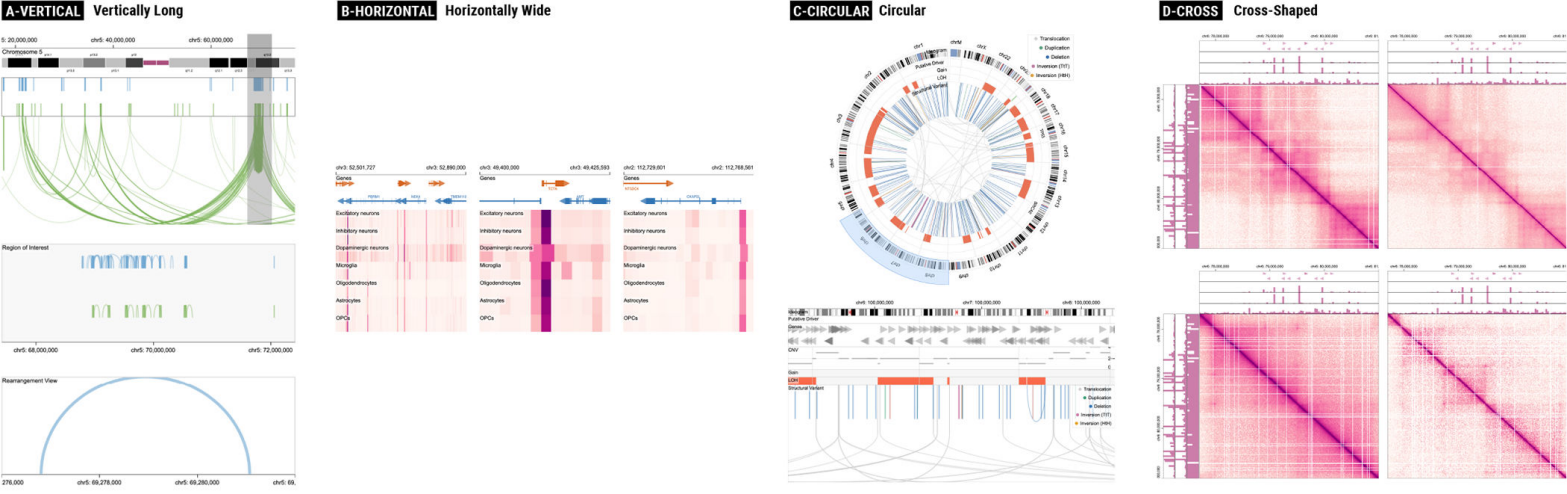
Examples of four typical composition patterns reproduced using Gosling [42]: (A) Three track groups with different levels of details [48], (B) side-by-side track groups with multiple genomic locations of interest, (C) circular overview with an additional linear detail view, and (D) four comparative matrices that compose multiple bar charts on the top and the left of each matrix.

Before discussing the usability issues of genomics visualization tools, we first classify their patterns for composing multiple tracks. Having a better understanding of composition patterns will enable a more systemic analysis since usability issues of multi-track visualizations often stem from their composition patterns. We classify track composition patterns into four exclusive categories: *vertically long*, *horizontally wide*, *circular*, and *cross-shaped* compositions. Adopted from the idea of text variant visualizations [24], we visually summarize the variants of track composition patterns in Fig. 4 which cover 92.5% of all surveyed tools. To demonstrate how actual visualization using these composition patterns looks like, we show examples for the four patterns in Fig. 5. Using the tiling algebra [68] (Sect. 4.1), we considered each track composition pattern of a tool as a sequence of tracks and tracks groups (operand) and arrangement types (operator). These sequence variants are then merged into four categories, i.e., the union of all sequences per category. The details about how to read the summary visualization (Fig. 4) are described in the caption.

#### Vertically Long

A majority of genomics tools (70%) uses vertical juxtaposition only (Fig. 4A). The number of tracks juxtaposed in such tools in their default settings varies greatly, between two and 52 tracks. All tools in this category used linear layouts (i.e., using Cartesian coordinates to encode visual representations) and mapped genomic positions to the *x*-axis. Many of such tools (11 out of 28) provided only one track group as can be seen by the thickest edge in the second track group (Fig. 4A-2), but more than half of the tools (6 out of 11) allowed users to add an unlimited number of tracks. Some other tools used multiple track groups. For example, a comparative browser CEpBrowser [9] showed two main track groups (Fig. 4A-2 and A-4) with two additional track groups with overview ideograms (Fig. 4A-1 and A-3). Several other tools [48] used multiple track groups to provide multiple levels of scales (Fig. 4A-2, A-4, and A-5), such as showing the whole genome, a specific chromosome, and additionally a local region, simultaneously.

#### Horizontally Wide

Although rarely observed, one tool [19] uses a composition pattern that is opposite to vertically long tools, i.e., using serial juxtaposition as the primary arrangement for composing many track groups (Fig. 4B). In this tool, a small number of tracks are stacked in a track group while as many track groups as users want can be added horizontally, making the entire visualization horizontally wide. This tool is different from the vertically long composition in that it focuses on seeing multiple genomic locations (e.g., genes of interest), while vertically long tools focus more on seeing a few number of genomic loci but with many tracks.

#### Circular

**Fig. 6.**
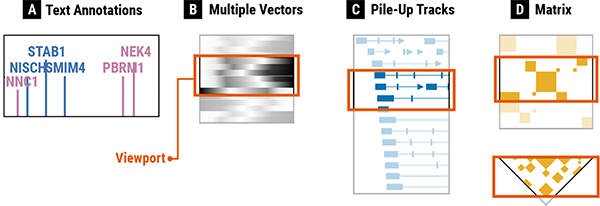
The visualization types with frequent usability issues: (A) visualizations with text labels, (B) visualizations based on multiple quantitative values, (C) Visualizations with piled up visual marks (e.g., gene annotation and alignment tracks), and (D) regular and rotated matrices.

Another set of tools (15%) contains circular tracks which make the composition patterns more complex than vertically long or horizontally wide composition. In our survey, all tools in this category showed only one circular visualization (Fig. 4C-1) that combines up to two track groups. Similar to track groups in vertically long tools, the most common composition patterns are stacking multiple tracks in parallel along the genomic axis (i.e., the thickest edge in Fig. 4C-2). As shown with the repetition representation (i.e., three dots and the grey ruler), users were able to add as many tracks as they want in several tools [20]. For the purpose of comparing multiple genomes, a subset of tools allowed composing at most two track groups into a single circular shape using either serial juxtaposition (Fig. 4C-3) and parallel juxtaposition (Fig. 4C-4). Several tools showed linear tracks, in addition to circular tracks. In such tools, circular tracks are served as overviews (i.e., showing larger regions in the genomic axis) while additional linear tracks are showing more local genomic regions (Fig. 4C-5, C-6, and C-7) [37].

#### Cross-Shaped

Another category of unique composition patterns stem from the use of ‘2D tracks,’ i.e., tracks that encode two genomic axes using both the *x*- and *y*-axes, such as matrix visualizations [29, 56]. In such tools, multiple 1D tracks can be juxtaposed on a 2D track in either two (i.e., leftward and upward) [56] or all four directions [29], providing contextual information on the two genomic axes. Multiple of such cross-shaped track groups are then composed in an either flow layout [56] or grid layout [29].

### 4.5 Usability Issues by Composition Patterns

**Fig. 7.**
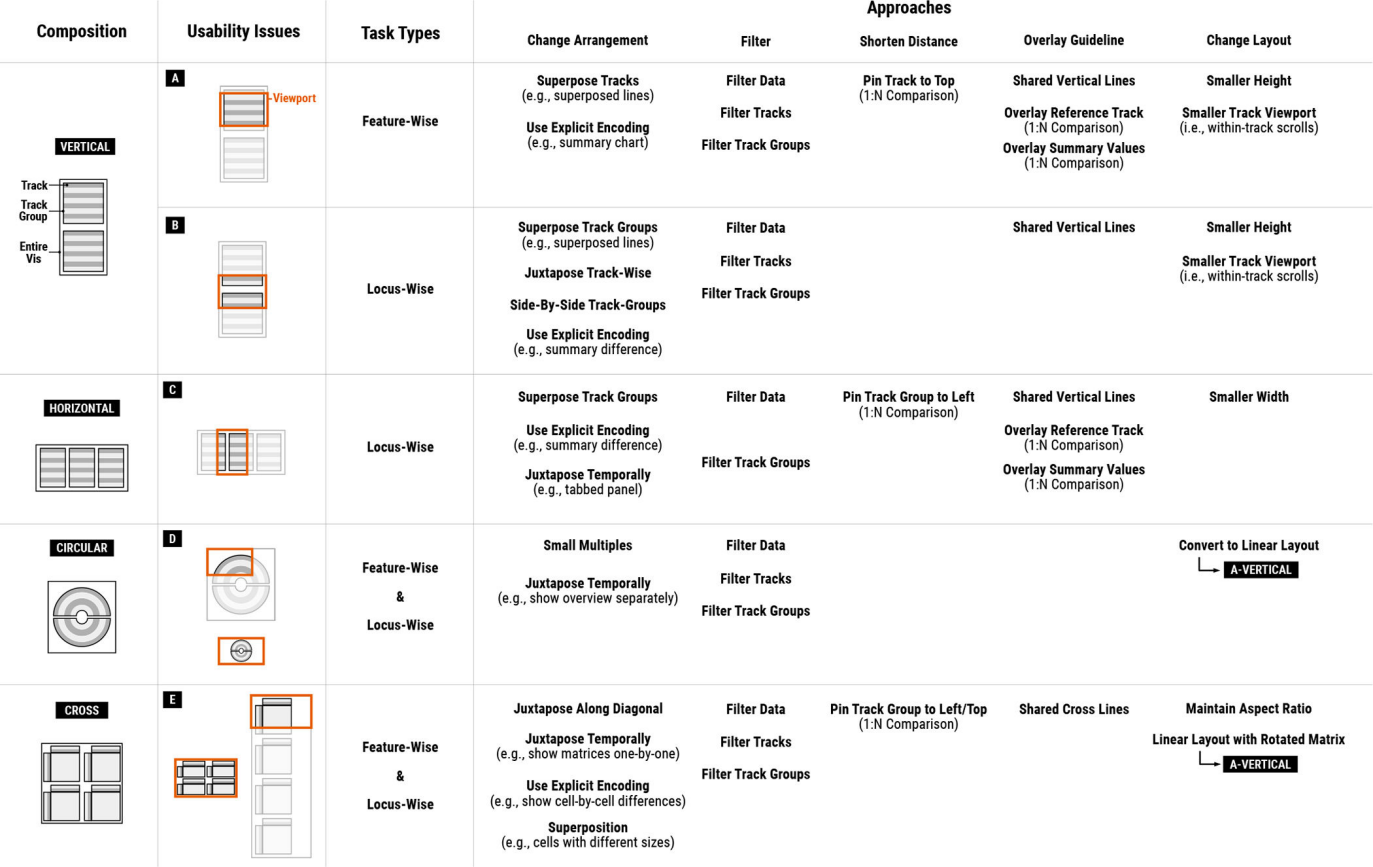
The summary of approaches to address usability issues of multi-track genomics visualization in smaller screens. These approaches are structured by track composition patterns (first column), observed usability issues (second column), and types of comparison and summarization tasks (third column) that are related to the corresponding composition category and usability issue.

Based on our composition pattern categories, we identify the usability issues. The five common usability issues are shown in Fig. 3. Also, these issues in different visualization types and composition patterns are illustrated in Fig. 6 and Fig. 7, respectively. These categories were initially taken from a survey on mobile visualizations [67] and are extended to reflect the structure of genomics visualizations in our survey (Sect. 3): an “out of the viewport” category has been extended for three components, i.e., track, track group, and visualization. These categories include (1) track out of the viewport, (2) track group out of the viewport, and (3) visualization out of the viewport, which refer to issues where individual tracks, track groups, and entire visualizations, respectively, become larger than the viewport and cannot be displayed within a screen. Another category is (4) unreadable visual marks, i.e., marks becoming too small to interpret, which was observed in many tools without any support of responsive layouts (e.g., resizing visualizations depending on resolutions). The last one is (5) visual occlusions (i.e., multiple marks being overlapped on top of each other) which was most frequently observed with text labels.

These five usability issues are related to two main factors that are frequently discussed in multi-track visualization design [41]: **display scalability** [12] and **effectiveness** of performing tasks. The first three categories (i.e., out of the viewport) are directly related to the display scalability while all five categories are related to the effectiveness. For example, tracks in a track group are intended to be explored together (e.g., seeing gene annotations to gain contextual information while browsing multiple experimental samples using bar charts). However, since such information cannot be displayed within a screen, users have to rely on their working memory to retain information from distant, no longer visible tracks (e.g., scrolling vertically and/or horizontally). This results in inaccurate and inefficient analysis [18]. For example, one tool [9] stacked two comparative track groups each of which contains over than 20 tracks (Fig. 7A–B). As a result, performing comparison tasks between track groups becomes challenging as users have to use vertical scrolling for a long distance (Fig. 7B). While it would be desirable to allow users to interactively adjust the compactness of visualizations, a majority of vertically long tools did not support resizing tracks or track groups at all (18 out of 28). If tools force users to use horizontal scrolling, in addition to vertical scrolling, due to the out of the viewport issues, the overall usability of a tool decreases [38]. The main issue of horizontally wide tools is when looking across multiple track groups, e.g., inspecting similar features around multiple genes of interest (Fig. 7C). One of typical usability issues for circular and cross-shaped composition tools is that adding a small number of tracks often makes individual tracks very tiny in smaller screens, leading to readability issues (Fig. 7D bottom and Fig. 7E left).

### 4.6 Visualization Types with Frequent Usability Issues

Identifying usability issues that are specific to track types can be helpful for visualization designers when they want to create tracks that should work properly across different resolutions. In our survey, some visualization types consistently showed usability issues in smaller screens (Fig. 2E and Fig. 6). The tracks that heavily use text annotations, such as lollipop plots [20], frequently led to visual occlusions (Fig. 6A). Some track types were vertically too long to fit on a screen in mobile devices, i.e., track out of the viewport (Fig. 6B–D). A set of such examples is tracks that visualize multi-dimensional quantitative values, such as heatmaps [19] or segregated bar charts [11] (Fig. 6B). Another set is track types that piles up glyph representations vertically, such as transcript annotation tracks [66] and read-level alignment tracks [55] (Fig. 6C). In some extreme cases, these pile-up tracks became much larger than the viewport as they try to pile up all visual elements in the given narrow screen space [66]. Other tracks that are commonly not shown within a screen includes regular matrices [56] and rotated matrices [39] (Fig. 6D).

### 4.7 Comparison to Compositions Outside Genomics

In this section, we compare our survey results with a survey of track composition patterns outside genomics [10]. This comparison can reveal the unique challenges of responsive designs for genomics visualizations. We found that the nature of multi-view composition patterns is quite different between genomics and non-genomics visualizations. When we compare our survey results with a survey on general multi-track visualizations [10], we find discrepancies in two main aspects: the number of tracks and frequent arrangement types.

In their survey, the majority of multi-track visualizations “presents less than five [tracks]” [10]. Genomics visualizations, however, contained more tracks on average by default (i.e., 8.1) while users are able to add as many additional tracks as they want in many tools (21 out of 39). According to our survey, the majority of the genomics visualizations (55%) provided more than five tracks by default. The track arrangement types observed in the two surveys are quite different as well. In their survey, horizontal juxtaposition was the most popular arrangement while using only vertical juxtaposition was one of the least frequently identified composition patterns. In contrast, our survey shows that vertically long composition was the most popular one.

These unique composition patterns of genomics visualizations seem to stem from the properties of genome-mapped data. For example, because of the multi-modal nature of genomics data [42] (i.e., visual patterns appear in multiple datasets), genomics experts seem to stack many visualizations on the same genomic axis and make them coordinated (i.e., supporting synchronized navigation), enabling the analysis of a particular genomic region based on multiple features. These unique patterns, i.e., many-view compositions, in genomics visualizations challenge the support of responsive designs due to the limited scalability of screen resolution in smaller screens.

## 5 Responsive Multi-Track Visualizations

In this section, we first identify low-level tasks of individual composition patterns based on a task taxonomy of genomics visualization [47]. We then distill approaches to address usability issues of composition patterns in terms of their display scalability and effectiveness. The tasks that are relevant to composition patterns, as well as proposed approaches, are illustrated in Fig. 7.

### 5.1 Task Identification

Nusrat et al. [47] identified seven genomics visualization tasks based on an earlier, general task taxonomy [6]. Since we focus on multi-track visualizations (i.e., using “multiple feature sets”), two low-level tasks are most relevant to our study: comparison and summarization. As we are mainly interested in supporting multi-track tasks (i.e., tasks that involve multiple tracks), we did not include other common tasks that are taken on a single track, such as Locate and Identify. In our study, we classify these two tasks into four sub-tasks considering their targets, i.e., features and genomic loci. For example, **feature-wise comparison** refers to comparing across multiple datasets (e.g., tracks in the same track group) while **locus-wise comparison** refers to comparing between genomic locations (e.g., multiple track groups that display different locations). Similarly, **feature-wise summarization** and **locus-wise summarization** mean summarizing information from multiple datasets or across multiple genomic locations. These four sub-tasks can be further classified based on the number of targets involved in tasks [17]. For example, 1:1 feature-wise comparison refers to comparing a pair of datasets, and 1:N locus-wise comparison refers to comparing a single genomic location of interest with multiple other locations. In summarization tasks, the number ‘N’ corresponds to the number of datasets or locations that are involved in for summarizing information.

Each composition pattern seems to serve different sets of tasks. For example, most of the vertically long tools provided only one track group, showing a single genomic location at a time. This makes the feature-wise comparison and summarization more appropriate while locus-wise tasks become challenging since multiple locations cannot be displayed at once in smaller screens. On the other hand, horizontally wide tools are more appropriate to show multiple genomic locations, enabling locus-wise tasks more effective. According to the design of existing circular and cross-shaped tools, the number of task targets seem to be more limited than other two sets of tools as our survey showed that less number of tracks and track groups can be displayed at once. This restricts users to perform many-target tasks (e.g., many-to-many comparison tasks). Consistent to this insight, circular tools in our survey only allowed at most two track groups for comparative analysis (e.g., two species in parallel arrangement [33]).

Appropriate tasks will vary depending on the screen resolutions. For example, users with smaller screens would not expect to perform the same tasks as in large screens. Therefore, controlling target tasks between screen resolutions is one of key factors when designing responsive visualizations. For example, a study of automating responsive designs [32] considered controlling the information granularity as one of the key aspects.

### 5.2 Multi-Track Responsive Designs with Examples

Based on composition patterns, usability issues, and tasks that we identified, we distill a set of responsive designs that can address the limited responsiveness of real-world genomics visualizations. To identify approaches, we reviewed taxonomy papers in the relevant areas: responsive designs [22, 30–32], multi-track compositions [10, 17, 18, 26, 41], and genomics visualization [47]. From existing responsive design patterns, we found two high-level categories that can directly affect the responsiveness of multi-track visualizations: changing arrangement between tracks (“Serialize Layout” [30]) and changing layout of tracks (e.g., “Reduce Width” and “Transpose Axes” [30]). We further expanded design patterns in these two categories using comprehensive taxonomies of multi-track composition (e.g., juxtaposition, superposition, explicit-encoding) [10, 17, 18, 26, 41] and genomics visualizations (e.g., circular layouts) [47]. Lastly, we took three design approaches that visualization designers commonly used for improving the effectiveness and display scalability of multi-track visualization [41] (i.e., filtering, shortening distance, and overlaying guidelines). As a result, we identified five high-level design categories: changing arrangements, filtering, shortening distance, overlaying guidelines, and changing layouts. Note that we did not include various responsive designs that are specifically for a single track (e.g., Encoding in [30]) since we focus on multi-track responsive designs and there already exist many responsive design examples that can be applied to a single track [22, 30]. The last five columns of Fig. 7 show design examples of individual categories in the context of genomics visualization. Since the design space for each category is extensive, we mainly focused on including examples that are most familiar in genomics visualizations [47] while other novel and more advanced techniques [44] can be also considered.

#### Change Arrangement

**Fig. 8.**
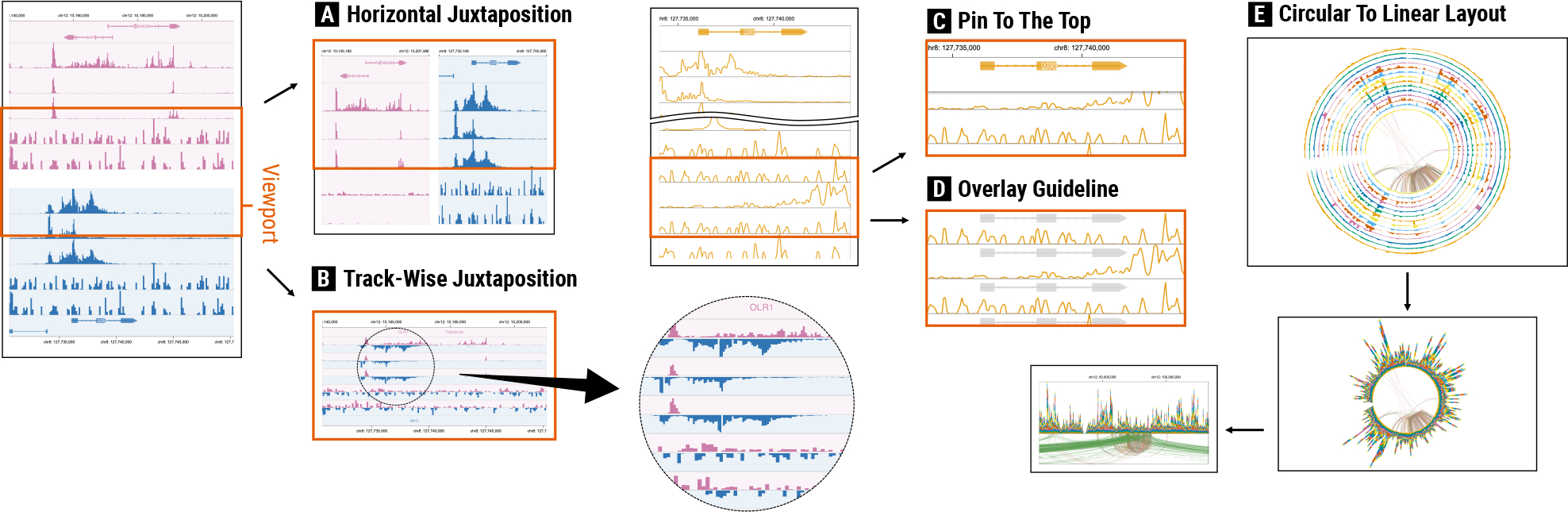
Examples of responsive multi-view visualizations implemented using the extended Gosling genomics visualization grammar [42]. Vertically long visualization with two track groups can use (A) horizontal juxtaposition for 1:1 locus-wise comparison or (B) track-wise juxtaposition to support N:N feature-wise comparison. For 1:N feature-wise comparison in a vertically long track group, (C) the reference track (e.g., gene annotations) can be pinned on the top to more effectively perform tasks by closely positioning target tracks for comparison. (D) Such information can be instead overlaid on the background of other tracks. (E) Circular visualizations with multiple bar charts can be converted into stacked bar charts to save space, which eventually use linear layout instead in a smaller screen.

**Fig. 9.**
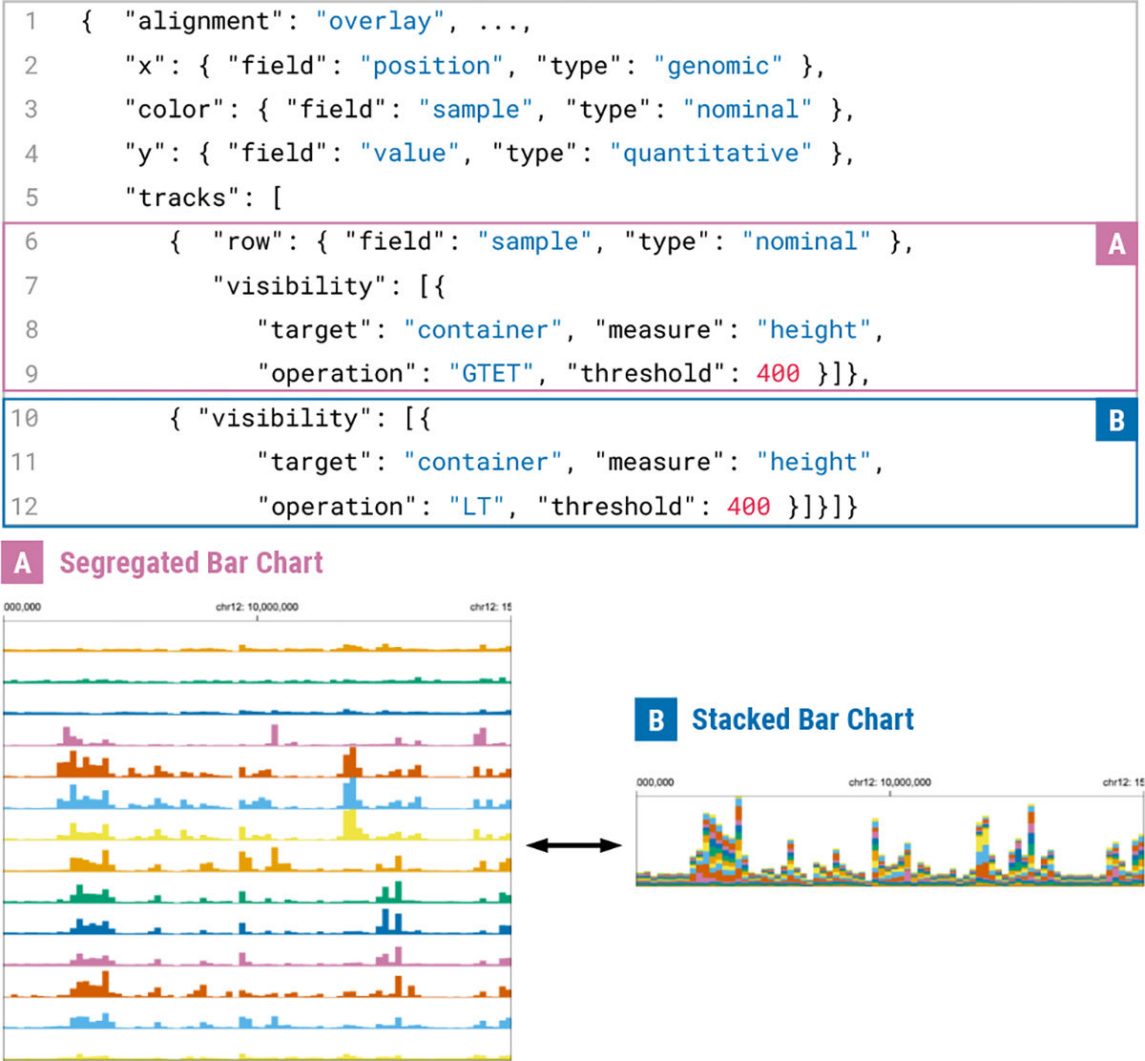
An example of track-level responsive designs with a spec (top) and a corresponding visualization (bottom). A segregated bar chart switches to a compact visualization, i.e., stacked bar chart, in a smaller screen, focusing on showing the hotspots on the genomic axis.

**Fig. 10.**
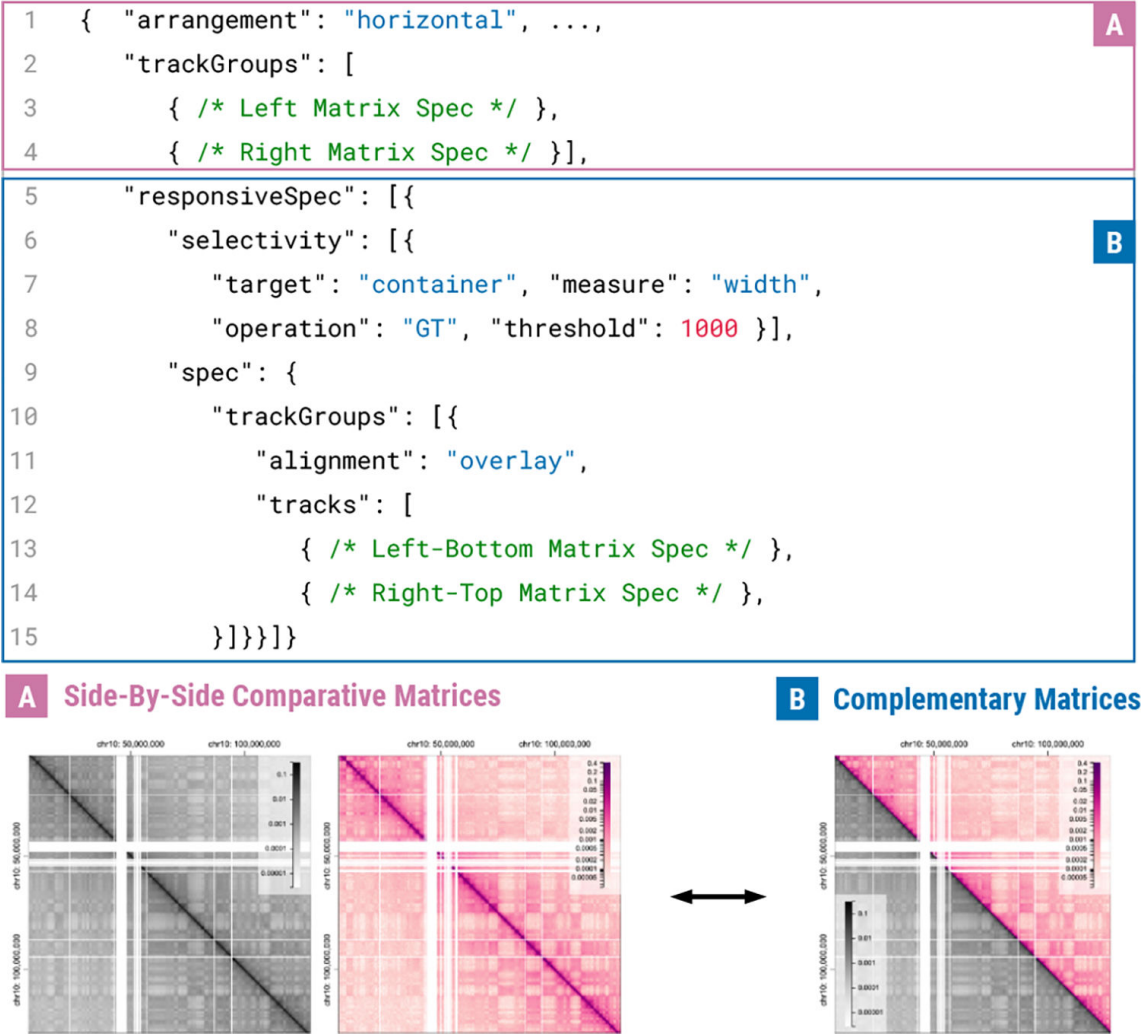
An example of track group-level responsive designs. Comparative matrices can be converted into a compact visualization in smaller screens, called complementary matrices [40].

Since the arrangement of tracks and track groups itself highly controls the balance between scalability and effectiveness of multi-track visualization [41], the most dramatic way to support responsive design would be to change the arrangement. There are several key arrangement options [10, 18, 41], including variants of juxtaposition and superposition, as well as combining multiple tracks into a single track to explicitly encode summary information (i.e., explicit-encoding). For example, in vertically long tools, multiple tracks of certain track types can be combined in smaller screens, such as using superposed line charts or stacked area charts [27] (Fig. 9), compressing the height of the track groups while still allowing users to see overall distributions. To support 1:1 locus-wise comparison between two track groups in vertically long tools, one can simply change the vertical arrangement of the groups to the horizontal one (Fig. 8A). To support N:N feature-wise comparison between a pair of track groups (e.g., CEpBrowser [9] and Xena [19]), designers can use track-wise juxtaposition, adopted from the idea of item-wise juxtaposition [41], i.e., directly juxtaposing corresponding tracks of two groups while supporting coordinated zooming and panning per track group (Fig. 8B). Since circular tools are not able to show a large number of track groups in smaller screens in our survey, designers can separate track groups into multiple circular visualizations, i.e., small multiples. For comparative matrices (i.e., cross-shaped tools), designers can render only one side of a diagonal in individual symmetric matrices and juxtapose them along the diagonal to save screen spaces without sacrificing task performances [40, 64] (Fig. 10). Designers can instead choose to combine multiple features in many tracks and show a visual summary using explicit-encoding, e.g., cluster a multi-row heatmap and then show cluster centroids.

#### Filter

Designers can control the amount of information shown in a visualization to serve more focused tasks in smaller screens in a scalable way. This may not directly improve comparison and summarization tasks but can handle scalability issues that we commonly found in the survey (i.e., out of the viewport). This filtering can be applied not only to data records [30] but also tracks and track groups. Our survey results showed that certain track types, such as read-level alignments and many-sample heatmaps, were not often visualized within a screen (Fig. 6C). By excluding data records using certain criteria (e.g., filtering out poor-mapping reads or low quality samples), designers can make the track more compact on smaller screens. Designers can also exclude certain tracks and track groups entirely to address the out of the viewport issues in vertically long and horizontally long tools or readability issues in circular tools. For example, designers can select certain tracks that are less important and can be excluded in smaller screens. All three methods (i.e., filtering data records, tracks, and track groups) can be used in all composition patterns, but filtering tracks in horizontally wide tools does not address the observed scalability issue since filtering tracks only makes the height of a track group compact and not the width (i.e., missing “Filter Tracks” in Fig. 7).

#### Shorten Distance

By shortening the distance between tracks, comparison and summarization tasks can be made more effective. Given that some composition patterns make the entire visualization much larger than the viewport on smaller screens, leading to scrolling for large distances, this approach can be useful for allowing users to perform tasks in a more effective way. This can be especially useful for 1:N comparison tasks in vertically long and horizontally wide tools. For example, designers can pin a certain track on the top of the viewport in vertically long tools (e.g., gene annotations or reference experimental sample track), similar to the “Freeze” feature in Google Spreadsheet [14], so that users can bring other tracks of interest closer to the pinned track (Fig. 8C). In horizontally wide tools, a track group of a certain genomic location can be frozen to the left for the similar purpose.

#### Overlay Guidelines

By overlaying certain information of interest directly in tracks, comparison and summarization tasks can be made more effective [41]. The simplest example is to add rules across tracks (e.g., rendering shared vertical lines in all tracks) so that users can more accurately relate genomic regions with information in other tracks. Designers can highlight more explicit information of contextual genomics regions (e.g., exons) as a background [69] to support more accurate 1:N feature-wise comparison tasks (Fig. 8D). Also, important summary information can be overlaid as well, such as mean or cumulative values of all samples.

#### Change Layout

Altering the layout of tracks and track groups (e.g., their sizes and shapes) can address usability issues in smaller screens. Any designs presented in a taxonomy [30] can be considered as potential approaches (e.g., resize or rotate tracks), as well as unique layout options in genomics visualizations (i.e., circular and linear layouts). For example, for some track types that often become very long in smaller screens compared to the height of the viewport (e.g., gene-related tracks [66]), designers can reduce the height of the viewport and rather support vertical scrolling in a track. This way, tracks with extreme sizes in smaller screens do not dramatically increase the height of entire visualization. Since circular tools are less scalable when it comes to increasing number of tracks and track groups on smaller screens, designers can convert the visualization into linear layouts [42] (Fig. 8E), similar to the responsive design found in a web article [7]. This results in converting the composition pattern into vertically or horizontally long compositions.

Some of these approaches can be used in combination. For example, filtering can be applied before using any other approaches to improve scalability. Similarly, layouts of tracks and track groups can be adjusted before and after applying any approaches. However, overlaying guidelines unavoidably leads to visual clutter, so multiple options in this category cannot be used at once.

### 5.3 Implementation in Gosling

We extend the Gosling visualization grammar for genomics data [42] to show how aforementioned responsive multi-track designs can be seamlessly supported in visualization grammars. Given the similarity between Gosling and other visualization grammars (e.g., Vega-Lite [57] and ggplot2 [65]), the concepts of our implementation can be adopted in other grammars as well. An alternative way to enable responsive designs in Gosling would be using imperative programming (e.g., CSS or dedicated JavaScript APIs). However, this will force users to maintain source codes in multiple forms (e.g., JSON for the grammar and CSS for the responsive design) and make the functionality language-dependent (i.e., seamless support of the same functionalities in the Python package of Gosling [43] is not possible).

To extend Gosling, we considered three main design rationales. Consistent with the concept implemented in the original grammar, the enhancements should be (1) expressive enough to support the wide-range of designs that we discussed in the paper while allowing (2) concise specification. Another important rationale is (3) learnability. Since the target audience of Gosling is not necessarily visualization experts, we need to make the responsive features seamlessly supported in Gosling without forcing users to learn an additional grammar. Motivated by these goals, we conceptualized responsive multi-track design into two main components: track-level and track group-level responsiveness.

#### Track-Level Responsiveness

Conceptually, track-level responsive designs can be considered as defining multiple visual representations in a single track and controlling their visibility conditions based on the context of devices (e.g., screen resolutions). Using this concept in the extended Gosling, users can, for example, define a segregated bar chart (Fig. 9A) that eventually becomes a stacked bar chart (Fig. 9B). This can be done by overlaying the two tracks (Line 1 and 5–18) and specifying visibility properties that determine when to show each track (i.e., depends on the height of the viewport in Fig. 9). Shared encoding specifications between the two overlaid tracks can be defined only once (Line 2–4) for conciseness. Using these approaches, users can flexibly define multiple levels of responsive designs in a single visualization (e.g., defining three visual representations for three sets of resolutions). Furthermore, users can display multiple visual representations at once in a single user-defined context (e.g., additionally showing text labels on a large screen on top of a base track).

#### Track Group-Level Responsiveness

Any other higher-level responsive design is achieved by using selectivity properties and defining alternative specifications. The compiler of Gosling checks if a certain selectivity condition is fulfilled (Line 7–19), and whenever the compiler finds a first condition that is fulfilled, the alternative spec (Line 14–18) will be overridden with the base spec (Line 1–5). For example, two comparative symmetrical matrices in a wide screen (Fig. 10A) can be changed into a more single complementary matrix [40] (Fig. 10B).

Since these approaches are conceptually controlling the selection of user specifications, an ability to express responsive designs is bound to the grammar. Since Gosling supports all four composition patterns as shown in Fig. 5, most of the approaches for the four different composition patterns in Fig. 7 are supported. An exception is pinning a track or a track group to the top and left.

## 6 Preliminary Expert Interview

To validate the potential usefulness and improvements of the Gosling extension, we conducted an interview with a genomics expert. He is a research scientist and has eight years of experience in the analysis of mutations in cancer genomes. One of his roles as a project manager is to create an interactive data portal^4^ that integrates interactive visualizations targeted for scientists and clinicians. He has neither participated in the design process of Gosling and its extension nor used Gosling previously. In the 80-min study, he was introduced to the concept of responsive designs and learned and used Gosling and its extension, followed by an interview. In general, he stated that supporting smaller screens is important for genomics visualization in a data portal: “In many hospitals, screens are very old, and this often relates to the amount of information that we can show. [The name of a front-end engineer] always thinks of how this interface looks like on small screens.” After learning the Gosling extension, he said that the concepts of switching representations are intuitive and that all responsive options he can think of seem to be supported in the Gosling extension. On the other hand, he commented on the dependencies of responsive features to Gosling: “This is sometimes confusing for me where on the hierarchy the responsive commands should be. It’s very connected to the rest of the grammar. That means you have to understand Gosling well first before you start. Intuitively, it would make sense.” The feedback matches our expectation that responsive features are considered as an advanced option in Gosling, but this also gives potential improvements to make the option more accessible (e.g., unifying the track-level and track group-level components).

## 7 Discussion

Comparison to Existing Techniques The main difference between the extended Gosling grammar and Cicero [31], a recent grammar for responsive design, is the approach to express alternative visualization designs. Cicero focuses primarily on relative difference, i.e., specifying what needs to be changed, while Gosling focuses on expressing individual visualizations for different resolutions. This makes the specification more concise in Cicero while requiring users to learn and remember supported options for this dedicated grammar. Gosling users, however, only need to learn the visibility and selectivity options and can apply their knowledge of the grammar for all other parts (e.g., defining an alternative specification using the Gosling grammar). However, this can sometimes result in a verbose specification if users want to define multi-level responsive designs. Given the trade-off between conciseness and learnability of these two approaches, we opted to emphasize learnability so as not to increase the barriers for the use of Gosling by genomics experts. Vega-Lite [57] supports a “Condition” property. It is not used in the context of responsive designs (e.g., checking screen sizes is not possible), but its syntax is similar to the Gosling extension in that it enables if-else assignments. Its main grammatical difference to Gosling, however, is that the condition is specified directly to individual properties, such as mark and channels (property-centric). However, Gosling enables defining responsive designs per target context (context-centric), which enables users to more easily see alternative designs at once for each context (e.g., Line 10–14 in Fig. 10). This is consistent to CSS where alternative properties can be defined together under a single @*media* group.

### Generalizability

In our study, we focused on genomics data since it is an important type of data that has become critical for a large number of applications in the biomedical field and is commonly analyzed using visualizations with multiple tracks. Despite having unique properties as discussed earlier (Sect. 4.7), genomics data and its visualization also share commonalities with other types of data and visualizations. For example, genomics data is similar to temporal data in that they both commonly use ordinal axes that represent many data points. Due to these properties, multiple time series data is often visualized by stacking multiple tracks vertically [35], making vertically long visualizations. By classifying the composition categories of their visualizations, designers can refer to approaches that we are suggesting here.

### Limitation

User interactions are another important aspect to consider when designing responsive visualizations [30] for different devices. Since user interactions has an enormous design space in multitrack designs [18, 34], we mainly focused on several important aspects, such as data, encoding, layout, and arrangement of multi-track designs, while not considering different input modalities in different devices at all (e.g., touch and mouse interactions). Follow-up studies to understand responsive interactions on multi-track visualizations could enhance our proposed framework.

## 8 Conclusion

In this paper, we report on a systematic survey of web-based multi-track genomics visualization tools in the wild (*N*=40). Our survey results revealed the lack of responsive designs which led to various usability issues in smaller screens. Combined with our comparison of survey results to the ones outside genomics [10], our results highlight both importance and challenges of responsive genomics visualizations. We categorized track composition patterns into four mutually exclusive groups (i.e., vertically long, horizontally wide, circular, and cross-shaped compositions) which are then linked with users’ analytical tasks (e.g., comparison and summarization). This expands the genomics visualization taxonomies [47] and can be useful for building task-based genomics visualization recommendation systems [50]. Based on the observed composition patterns, as well as the usability issues that stem from the composition patterns, we identified diverse responsive designs taken from existing visualization taxonomies and design guidelines. By extending the Gosling visualization grammar [42], we show how responsive designs can be supported seamlessly in existing visualization grammars. While we focused on genomics data, the overall workflow of our study, i.e., from the analysis of composition patterns, tasks, and usability issues to the implementation of responsive designs based on existing knowledge, can be adopted in other fields. Valuable future work would be automating responsive designs, such as changing layouts by balancing scrolling and information granularity. Also, we have focused on regular to small screens, and it would be interesting to explore responsive genomics visualization for large displays [3].

## Supplementary Material

Supplementary Material

Supplementary Video

## Footnotes

1 Based on Google Analytics for https://portal.hubmapconsortium.org/ and https://data.4dnucleome.org/.

2 As of March 17, 2022.

3
https://developer.chrome.com/docs/devtools/

4 Clinical Genome Analysis Platform (CGAP): https://cgap.hms.harvard.edu/
