# Supplementary material for: Multi-View Design Patterns and Responsive Visualization for Genomics Data
Source: IEEE Trans Vis Comput Graph. Author manuscript; Available in PMC 2023 Apr 5. (PMC10040461; doi:10.1109/TVCG.2022.3209398)
Supplement: Supplementary Material [file NIHMS1846028-supplement-Supplementary_Material.pdf]

Supplemental Material

This document provides the complete grammar of Gosling in an abstract code description adopted from Ren et al. (Ren, Lee, and Brehmer 2018). Please also refer to the online demos (https://gosling.js.org) to see the examples of actual JSON specifications.

Notation

Symbol	Meaning
:=	Assignment
*	Zero to more
|	Or
?	Optional
Number	Numeric value (e.g., 16)
String	String value (e.g., “green”)
Boolean	Boolean value (e.g., true)
[ ]	Array

Top-level Responsive Resizing

This determines whether to stretch either height, width, or both of the entire visualization to fit into its container component. If this option is used, entire tracks and track groups will be resized proportionally, based on their sizes specified in the specification.

```
root := responsiveResize?, trackGroup, ...
responsiveResize := Boolean | DimensionWiseResize
DimensionWiseResize := width?, height?
(width | height) := Boolean
```

Track-level Responsiveness

```
trackGroup := tracks, alignment?, responsiveSpec?, ... | trackGroup
alignment := 'stack' | 'overlay'
tracks := track[]
```

```
track := title?, width, height, assembly?, data, dataTransform?, mark,
     x?, y?, color?, size?, row?, text?, stroke?, strokeWidth?,
     opacity?, tooltip?, displacement?, visibility?
visibility := VisibilityCondition[]
VisibilityCondition := measure, threshold, operation,
     conditionPadding?
measure := 'width' | 'height' | 'aspectRatio'
threshold := number
operation := 'LT' | 'lt' | 'less-than' | 'GT' | 'gt' | 'greater-than'
     | ...
conditionPadding := number
```

Track Group-level Responsiveness

```
trackGroup := tracks, alignment?, responsiveSpec?, ... | trackGroup
responsiveSpec := alternativeSpecAndSelectivity[]
alternativeSpecAndSelectivity := spec, selectivity
spec := Partial<trackGroup>
selectivity := selectivityCondition[]
selectivityCondition := target?, measure, threshold, operation
target := 'self' | 'container'
measure := 'width' | 'height' | 'aspectRatio'
threshold := number
```
